# Definition of clinical immunology around the globe

**DOI:** 10.3389/fimmu.2025.1483391

**Published:** 2025-01-28

**Authors:** José C. Crispín, Tineke Cantaert, Alberto Pinzon-Charry, Domenico Mavilio, Ahmed Seri, Pierre Miossec

**Affiliations:** ^1^ Clinical Immunology Committee, International Union of Immunological Societies, Berlin, Germany; ^2^ Escuela de Medicina y Ciencias de la Salud, Tecnologico de Monterrey, Monterrey, Mexico; ^3^ Department of Immunology and Rheumatology, Instituto Nacional de Ciencias Médicas y Nutrición Salvador Zubirán, Mexico City, Mexico; ^4^ Immunology Unit, Institut Pasteur du Cambodge, Pasteur Network, Phnom Penh, Cambodia; ^5^ Queensland Paediatric Immunology and Allergy Service, Queensland Children’s Hospital, South Brisbane, QLD, Australia; ^6^ School of Science, Griffith University, Nathan, QLD, Australia; ^7^ Department of Medical Biotechnologies and Translational Medicine, University of Milan, Milan, Italy; ^8^ Unit of Clinical and Experimental Immunology, IRCCS Humanitas Research Hospital, Milan, Italy; ^9^ Clinical Immunology and Allergy Department, Soba University Hospital, University of Khartoum, Khartoum, Sudan; ^10^ Immunogenomics and Inflammation Research Unit, Hôpital Edouard Herriot, Hospices Civils de Lyon, Lyon, France

**Keywords:** allergic diseases, autoimmunity, autoinflammation, clinical immunology, hematology-oncology, inborn errors of immunity/primary immunodeficiencies, infections, international union of immunological societies/IUIS

## Abstract

Immunology has gradually become a core part of many medical specialties. Immune cells and immune mediators are now known to participate in the pathogenesis of a wide variety of diseases and therapies based on the modulation of immune function are increasingly used. Traditionally, clinical immunologists have studied patients with inborn errors of immunity (IEI), previously known as primary immunodeficiencies, and with allergic conditions. More recently, clinical immunology has become involved with a broader array of disorders. The Clinical Immunology Committee of the International Union of Immunological Societies set out to understand how clinical immunologists perceive their specialty to identify similarities and differences in training and practice around the globe. For this purpose, a specific questionnaire was designed and distributed amongst our member societies. More than 500 participants answered the questionnaire, 80% of whom had completed their training. Roughly two thirds of respondents were physicians directly involved in patient care. We found that though the number of diseases and processes in which immune mechanisms are involved has considerably grown, 90% of participants agree with the 1993 World Health Organization definition of Clinical Immunology. We propose that the increased complexity of the field opens a need for multidisciplinary teams of clinicians and basic researchers and for a broader training of specialists.

## Introduction

During the last couple of decades, our understanding of the immune system and its role in disease pathogenesis has enormously increased. We now know that the immune system is relevant for a large number of diseases, as it is involved in different steps of their development and expression (1). The immune system represents a ubiquitous regulatory system that maintains tolerance during steady state while sensing the presence of pathogens and cellular stress caused by different mechanisms (2, 3). That capability is translated into the generation of innate and adaptive effector mechanisms in response to a variety of cellular disease states. For instance, signals released from diseased, stressed, or infected cells can trigger immune activation, subsequently manifesting as chronic inflammation which in turn, affects disease expression. As a result, immune and inflammatory components have been described in most chronic diseases, even in those in which the immune system was originally not thought to be involved. Accordingly, chronic inflammation is now recognized as a key component of neurodegenerative diseases (4), cancer (5), cardiovascular diseases (6), and obesity (7).

The successful development of therapies based on the manipulation of the immune system has further reinforced our understanding of the role of the immune system in many diseases and has expanded and popularized knowledge about the immune system in fields dominated by experts not trained in immunology. For example, many clinicians now use monoclonal antibodies as neutralizing drugs or cytotoxic agents to treat their patients (8). In many cases the targets of these therapies are products released by the immune system (e.g., TNF or other cytokines) or cells of the immune system (e.g., B cells in patients who receive anti-CD20 antibodies). In some fields, autologous T cells activated, expanded, and engineered to express chimeric antigen receptors (i.e. CAR T cells), are now part of the therapeutic armamentarium (9). By blocking key components of the immune system, these therapies cause secondary immune deficiencies or immune dysregulation (10). As an unintended result of this rapid expansion, immunology has gradually seeped into the realm of a large number of medical specialties that previously did not consider the immune system or its mediators as relevant for their practice.

Clinical immunologists have traditionally studied diseases caused by defects in the immune system. For example, primary immunodeficiencies, now included under the broader definition of inborn errors of immunity (IEI), represent a group of hereditary conditions caused mostly by monogenic defects in genes that encode proteins with non-redundant functions in the immune system (11). IEIs have been traditionally studied and treated by clinical immunologists. However, during the last decades, the exponential growth of the number of IEIs as well as the increase in the types of diseases considered under the definition (i.e., not only susceptibility to infection but also autoinflammatory syndromes, disorders of immune dysregulation, primary atopic/allergic disorders, and even cancer susceptibility) has substantially broadened the clinical spectrum covered by clinical immunology. In addition to this, the conceptual expansion of immunology into many diseases and treatments has brought the specialty into a central position in clinical medicine.

These issues have raised concerns about the breadth of the practice of clinical immunology as they open the question of whether clinical immunologists must become involved in the large number of clinical scenarios in which immunology has become central. Our preliminary discussions with colleagues demonstrated that there is high heterogeneity in the definition of Clinical Immunology around the world. Moreover, training, evaluation, daily practice, and research may differ in different countries and even between universities of the same country. As an initial approach to this question, the Clinical Immunology Committee of the International Union of Immunological Societies (IUIS) set out to conduct a world-wide survey aimed at exploring how clinical immunologists around the globe define their specialty. In particular, whether they agree with the 1993 World Health Organization (WHO) definition of Clinical Immunology (12) and to assess the breadth of conditions addressed by Clinical Immunologists during their training and their practice.

## Materials and methods

### Questionnaire design and distribution

We designed a questionnaire to understand how Clinical Immunology is conceptualized in terms of practice and teaching in countries throughout the globe (**Supplementary Material**). We used Google forms to distribute the questionnaire and collected responses from March to October 2023. To this end, we contacted the IUIS and FOCIS, as well as local Immunology or Clinical Immunology Societies from Africa, America, Europe, Asia, and Oceania, and asked them to distribute the questionnaire to their members. We asked the different societies to include all their members, including trainees and professionals practicing Clinical Immunology.

## Results

### Questionnaire respondents

**Figure 1:**
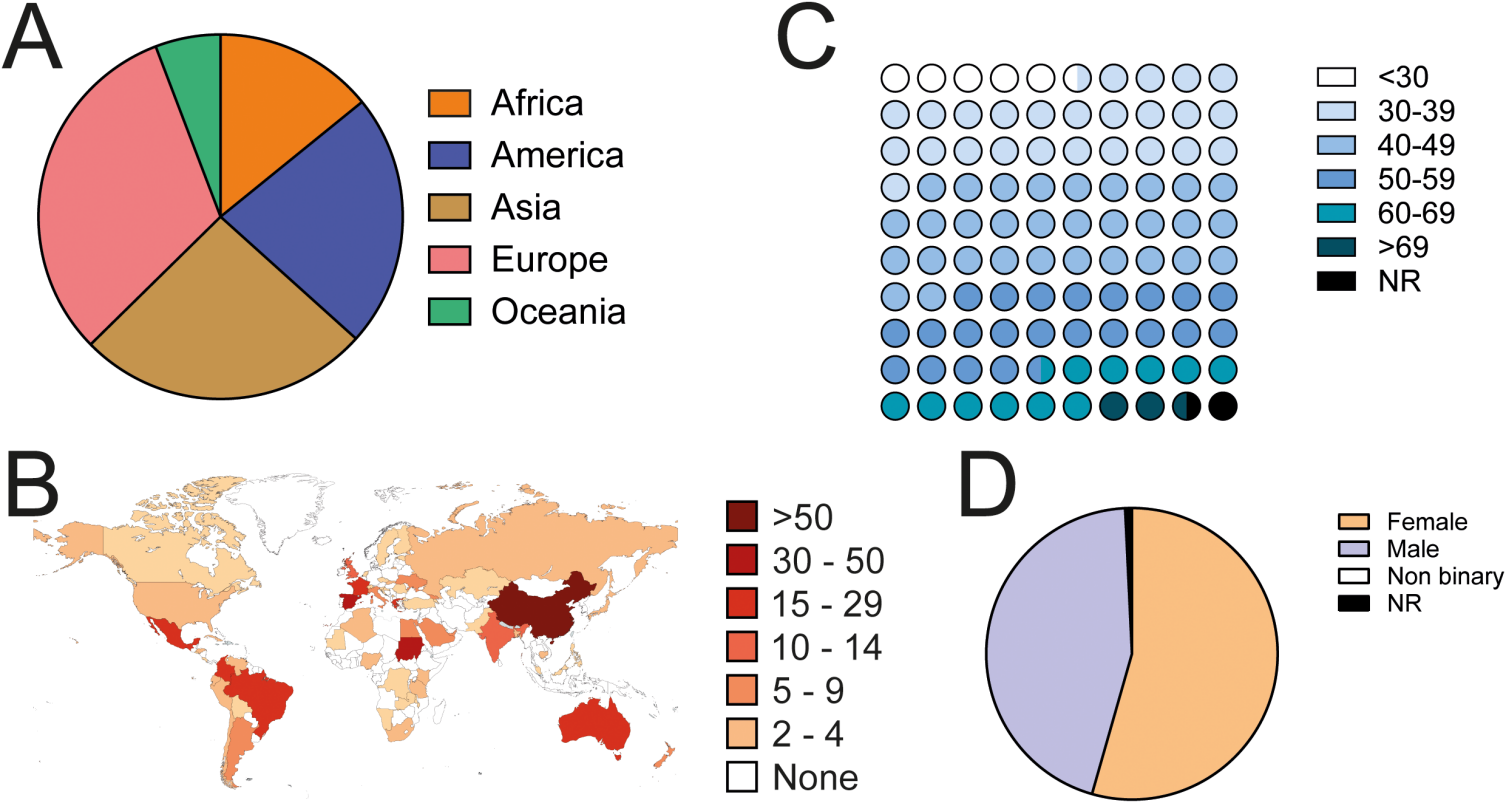
Geographical and demographic composition of responding clinical immunologists. **(A)** Distribution of respondents from different continents. **(B)** Number of respondents from each country. The map was generated using www.mapchart.net. **(C)** Age distribution of respondents. **(D)** Gender of respondents. NR, not responded.

**Figure 2:**
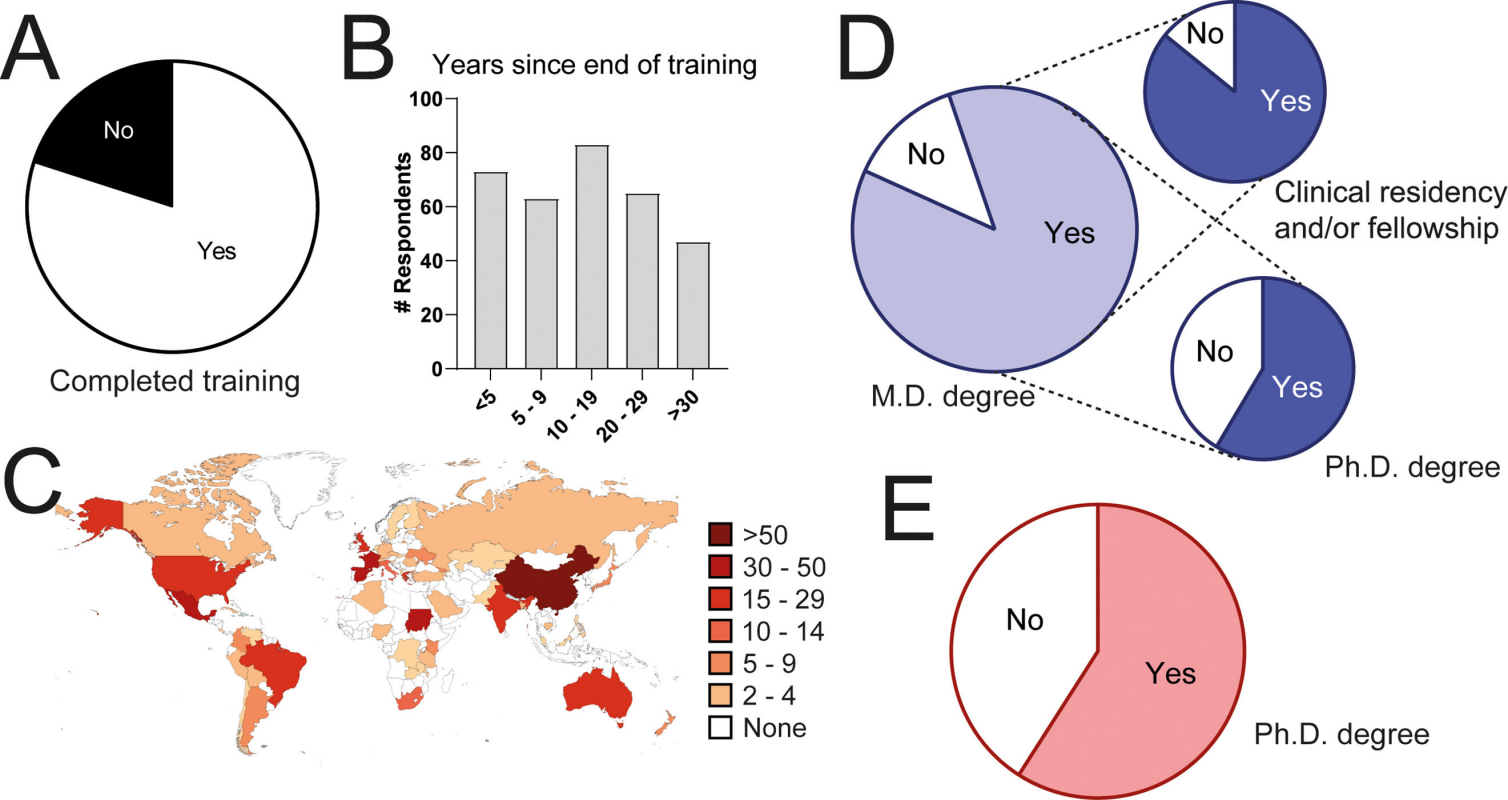
Training in responding clinical immunologists. **(A)** Percentage of respondents who had completed their training. **(B)** Years elapsed since the end of their training. **(C)** Countries where they trained. **(D)** Percentage of respondents with an M.D. degree or equivalent and fraction of them who did a residency or clinical fellowship, or who did a Ph.D. **(E)** Percentage of respondents with a Ph.D. degree.

We received 531 answers from clinical immunologists from 63 countries (**Figures 1A, B**). Respondents from all age groups participated (**Figure 1C**) and gender was balanced between males and females (**Figure 1D**). Eighty percent of respondents had completed their training at the time of answering the questionnaire (**Figure 2A**) and the time elapsed since was variable, from less than 5 years, to more than 30 (**Figure 2B**). They had trained in a large variety of countries, from all continents (**Figure 2C**). One-third (32.9%) had done all or some of their training in a country different from where they lived. Eighty-seven percent of respondents held an M.D. degree (or equivalent). Eighty-five percent of them had done a clinical residency and/or clinical fellowship and 58% held a Ph.D. degree (**Figure 2D**). Fifty-nine percent of all respondents had obtained a Ph.D. degree in an area related to immunology (**Figure 2E**).

**Figure 3:**
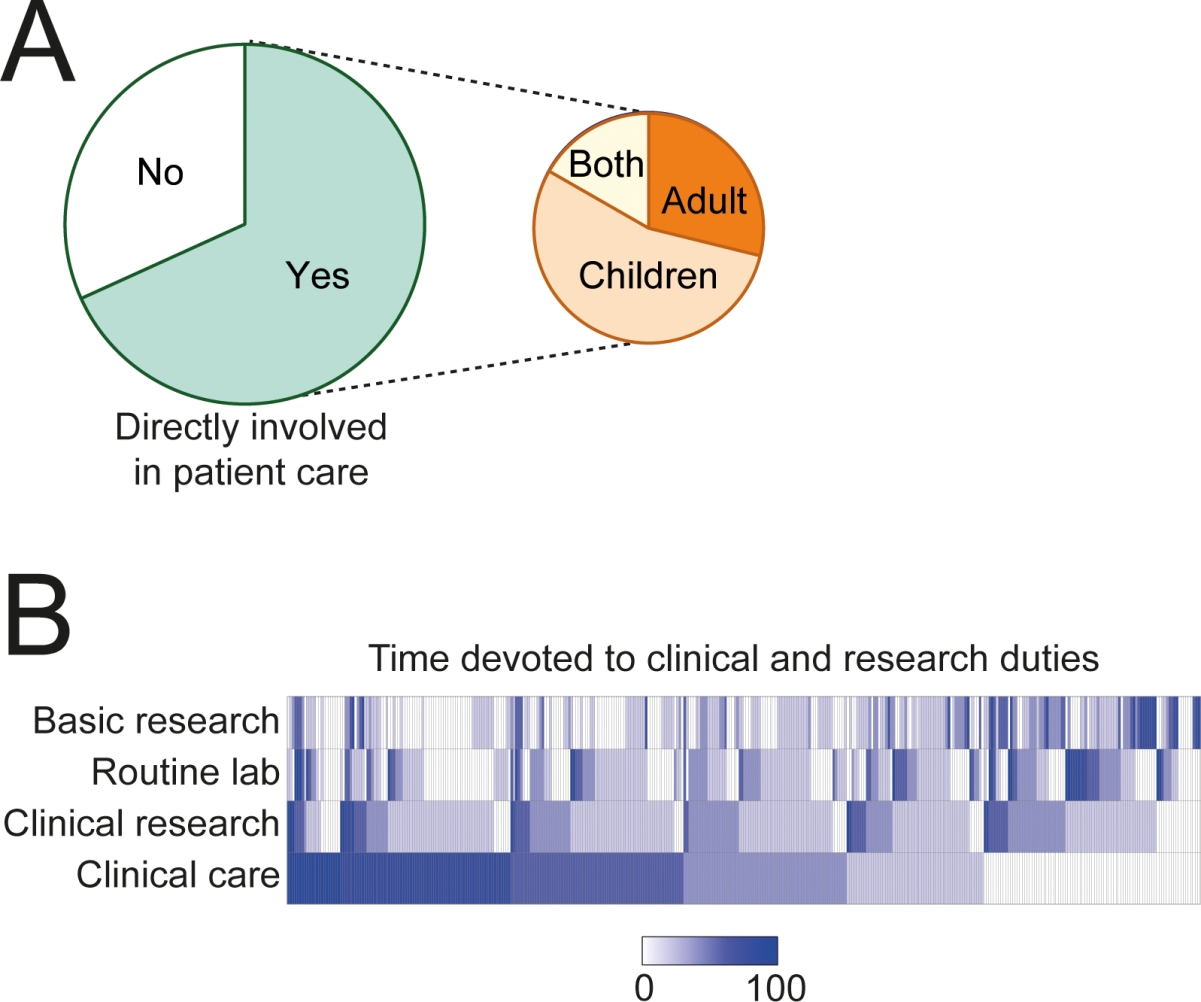
Professional profile of respondents. **(A)** Percentage of respondents involved in patient care. **(B)** Distribution of time (%) devoted to clinical care, clinical research, basic research, or routine lab. Each column corresponds to one respondent.

Sixty-eight percent of respondents stated they were directly involved in patient care. Roughly one-third of them exclusively consulted adults and one-half children (**Figure 3A**). When asked about time devoted to different activities, almost one-half of respondents reported spending more than 50% of their time in patient care, whereas time devoted to other duties, including clinical or basic research, or working in a clinical laboratory, were more variable among respondents (**Figure 3B**). In summary, the questionnaire was answered by a large number of clinical immunologists from around the globe, with a balanced representation of geography, ages, and gender.

### The definition of clinical Immunology according to the WHO

**Figure 4:**
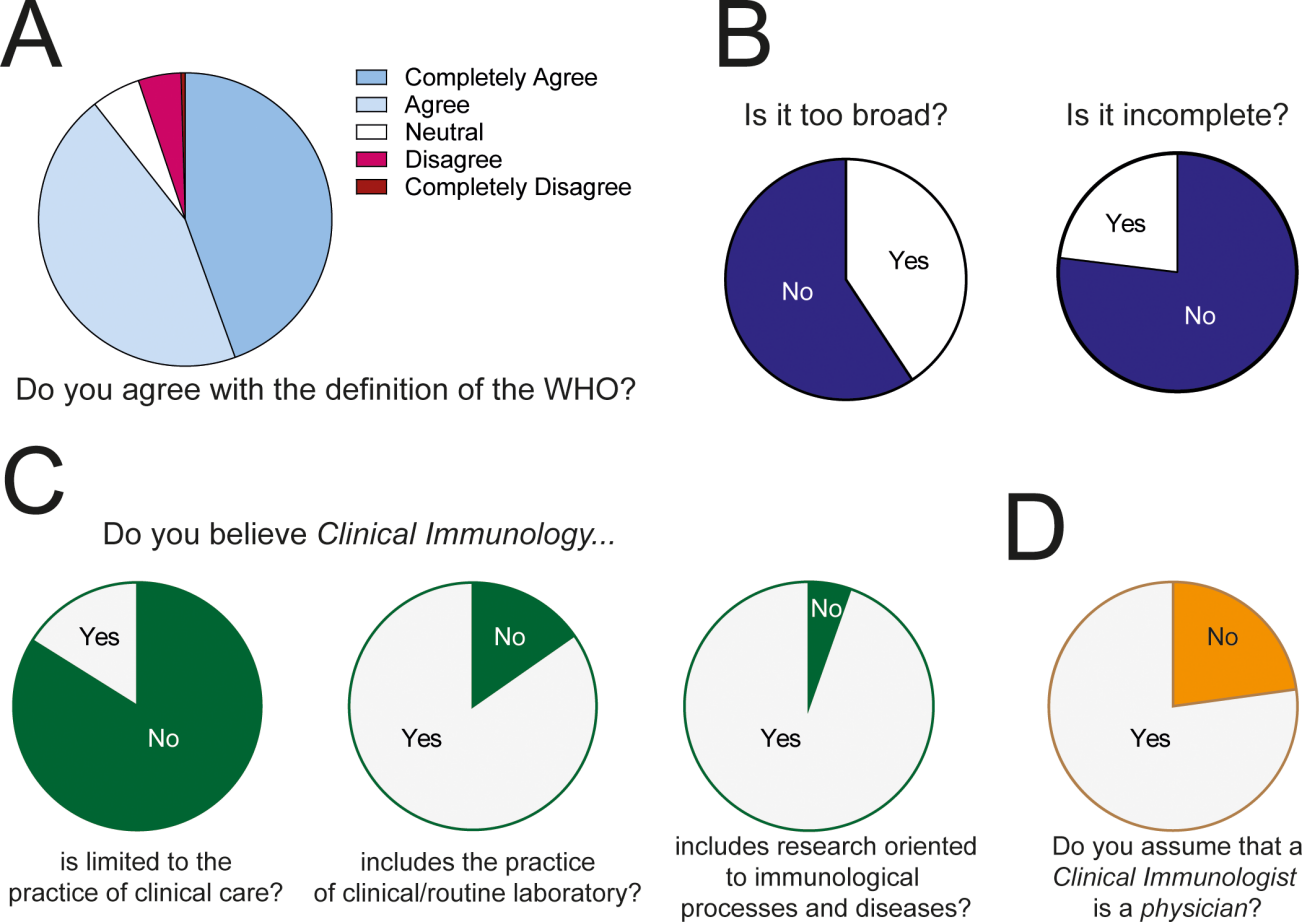
Definition of Clinical Immunology. **(A)** General opinion of respondents about the WHO definition. **(B)** Opinion about the breadth of the WHO definition of Clinical Immunology. **(C)** Opinion about the breadth of Clinical Immunology. **(D)** Opinion about the identity of a Clinical Immunologist.

The WHO defines Clinical Immunology as a clinical and laboratory discipline dealing with the study, diagnosis, and management of patients with diseases or disease processes resulting from disordered immunological mechanisms, and conditions in which immunological manipulation forms an important part of therapy and/or prevention (12). Ninety percent of respondents stated that they agreed with the WHO definition of Clinical Immunology. Five percent said they felt neutral about the definition and five percent disagreed (**Figure 4A**). Forty percent of them thought the WHO definition is too broad and 23% felt it is incomplete (**Figure 4B**). Most respondents (84%) believed that clinical immunology is not limited to the practice of patient care. Likewise, most thought that it includes clinical or routine laboratory practices (85%) and research oriented towards the study of immunological mechanisms and diseases (95%) (**Figure 4C**). However, 77% of respondents said that the term clinical immunologist refers to a physician who directly cares for patients (**Figure 4D**).

Collectively, the data shows that, in general, clinical immunologists agree with the definition of the WHO. However, an important fraction of them believes the definition to be too broad. Moreover, the generalized notion of a clinical immunologist is a physician whose primary occupation is treating patients, while conducting research or performing laboratory work, is regarded as secondary. This bias is probably related to the fact that most respondents are heavily involved in clinical care (**Figure 3B**) and thus their definition of a clinical immunologist may be skewed by their own professional profile.

**Figure 5:**
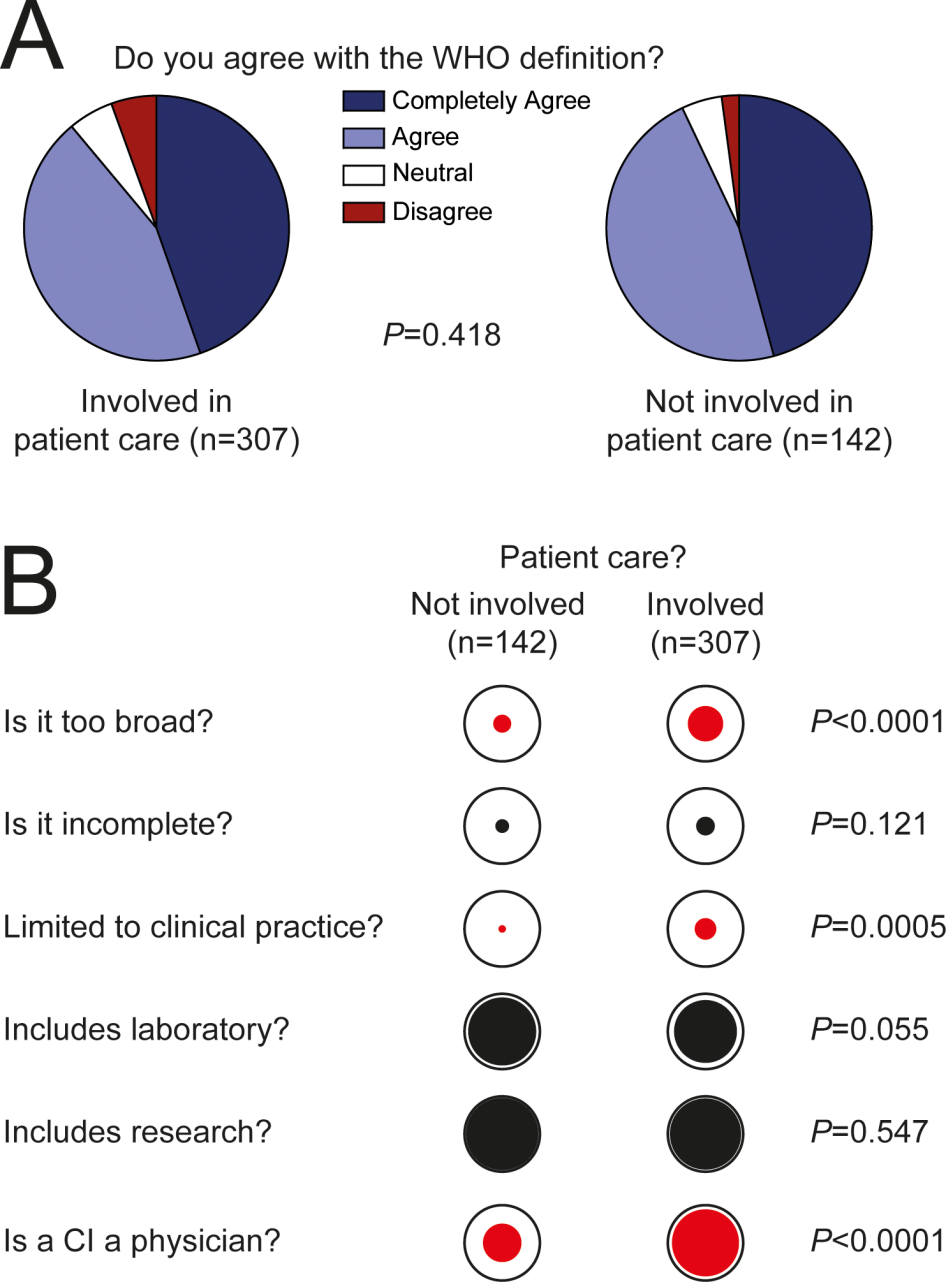
Conceptualization of Clinical Immunology according to professional profile. **(A)** Opinion of respondents that are directly involved in patient care and of those who are not, about the WHO definition. **(B)** Opinion of respondents about the WHO definition of Clinical Immunology and about the professional profile of a Clinical Immunologist. The diameter of the internal circles represents the proportion of positive answers. Red color indicates that the difference is statistically significant. Proportions were compared using Chi square.

To determine whether respondents directly involved in patient care conceptualize Clinical Immunology in a different manner than those who are not involved in patient care, we compared their responses (**Figure 5A**). Though a great majority of respondents from both groups (involved in patient care; n=307, vs. not involved in patient care; n=142) agreed with the WHO definition (**Figure 5A**), their opinions about some of its aspects were different (**Figure 5B**). More respondents directly involved in patient care thought the WHO definition is too broad (46.91% vs. 23.78%, *P*<0.0001) and that Clinical Immunology refers exclusively to clinical practice (28.62% vs. 9.88%, *P*=0.0005). A great majority of respondents involved in patient care assumed that a Clinical Immunologist is a physician (88.93% vs. 51.05%, *P*<0.0001). These results indicate that the conceptualization of Clinical Immunology is heavily influenced by the training of each respondent and their day-to-day professional activity.

### Practice and teaching of clinical immunology

**Figure 6:**
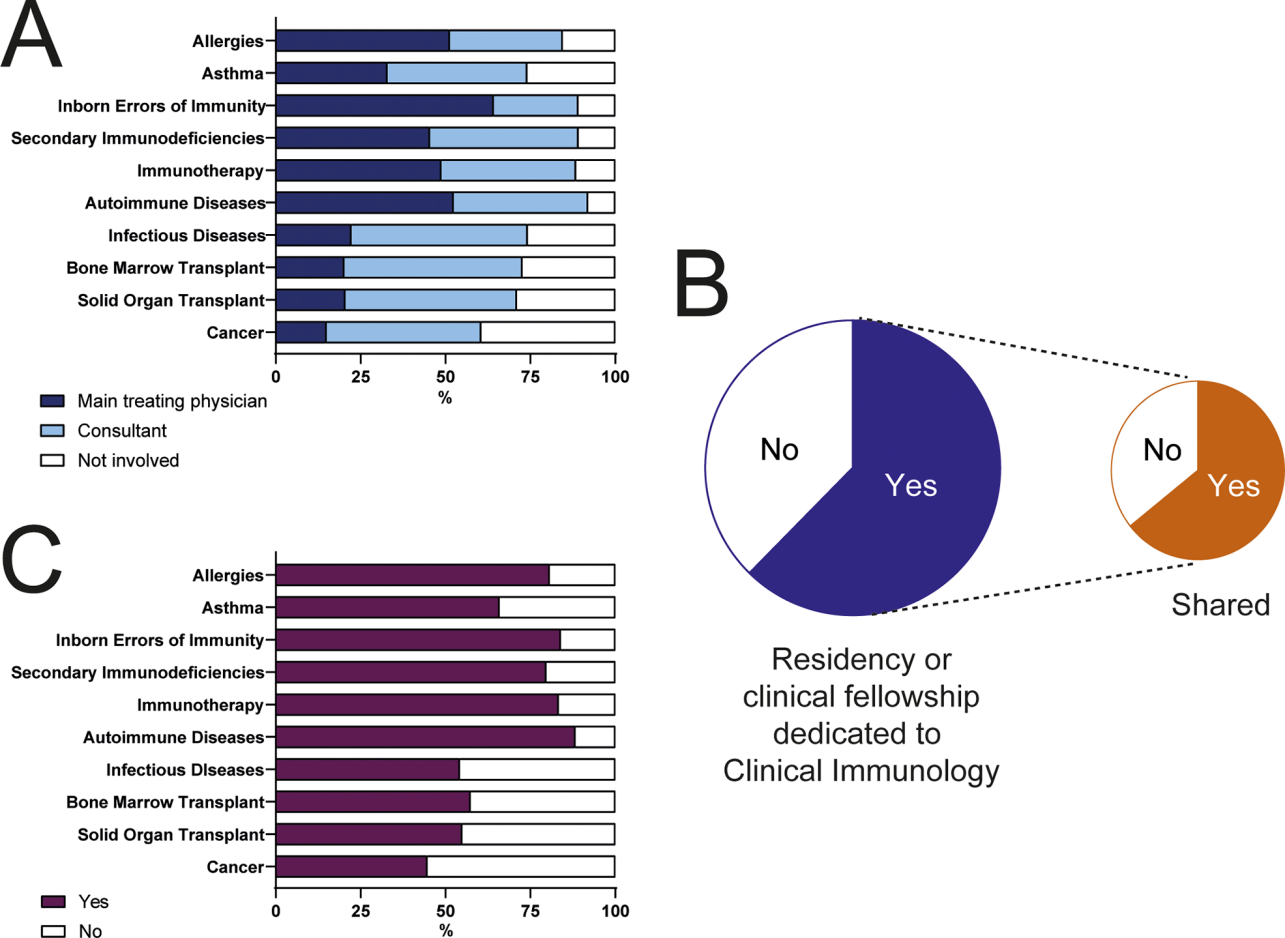
Conditions treated by Clinical Immunologists and taught during training of the specialty. **(A)** Respondents were asked whether in their countries, clinical immunologists acted as main treating physicians, as consultants, or were not involved in the management of patients with the enlisted conditions. **(B)** Percentage of countries that have a residency or clinical fellowship program dedicated to Clinical Immunology. **(C)** Conditions considered in the curricula of Clinical Immunology programs.

We inquired about the types of diseases that clinical immunologists treat in different parts of the world. To this end, we asked whether clinical immunologists were regarded as main treating physicians, consulting physicians, or were not involved in the care of patients with that type of condition. As shown in **Figure 6A**, inborn errors of immunity were considered by most respondents (64%) conditions in which clinical immunologists act as main physicians. Next, clinical immunologists were considered the main treating physicians of patients with allergic diseases, secondary immunodeficiencies, autoimmune diseases, and during immunotherapy in roughly 50% of countries. Finally, asthma, transplantation (bone marrow or solid organ), infectious diseases, and cancer were considered conditions in which clinical immunologists act most commonly as consultants or are not involved in patient care (**Figure 6A**).

Sixty-two percent of countries have a residency or clinical fellowship dedicated to the training of clinical immunologists. In 57% of the cases, the training program is not exclusively focused on clinical immunology, but shared, most commonly, with allergy (**Figure 6B**). Accordingly, immunodeficiencies, immunotherapy, allergies, and autoimmune diseases are included in the curricula of most clinical immunology fellowships (**Figure 6C**).

### Geographical heterogeneity

We compared the characteristics of respondents from different continents, to gauge their homogeneity (**Table 1**). Except for Africa, where a majority (73%) of respondents were female, in the rest of the continents, the male: female ratio was close to 1. In all continents, most respondents had an M.D. degree, from 72% in Asia, to 100% in Oceania, and most had done clinical immunology residencies or clinical fellowships (73% in Asia, to 93% in America). Figures for Ph.D. degrees were more variable. Around one third of respondents from Africa and Oceania held Ph.D. degrees, compared with more than 80% from Europe. The respondents from Africa had, on average, completed their training later than respondents from the rest of the world. Finally, less participants from Asia and Europe were directly involved in patient care compared to their peers in Africa, America, and Oceania (**Table 1**).

**Table 1:** Geographical origin of respondents.

	Africa	America	Asia	Europe	Oceania	Total	p
Number	61	95	88	136	24	404	
Male (%)	32.79	46.32	53.41	51.11	41.67	45%	0.02^a^
M.D. degree (%)	93.33	94.74	72.41	88.24	100	86.9%	<0.0001^a^
Residency or clinical fellowship (%)	77.05	92.63	72.73	83.09	83.33	80.9%	0.004 ^a^
Ph.D. degree (%)	31.15	46.32	61.36	82.35	29.17	59%	<0.0001^a^
Year when training was completed (mean ± SD)	2016 ± 6.22	2008 ± 11.53	2008 ± 13.22	2004 ± 11.07	2006 ± 12.32	2008 ± 11.57	<0.0001^b^
Directly involved in patient care (%)	88.52	94.74	42.05	67.65	100	68.2%	<0.0001^a^
>50% Patient care (%)	52.46	67.02	28.95	26.56	75	46.1%	<0.0001^a^

^a^ Chi square; ^b^ One-way ANOVA.

When we compared the opinions about the WHO definition of clinical immunology, we observed that more African (54%) and American (59%) clinical immunologists considered the definition too broad, compared with respondents from other continents (**Table 2**). Except for clinical immunologists from Oceania, 56% of which considered that clinical immunology refers exclusively to patient care, in the rest of the continents only 20 to 25% of respondents shared that opinion. The great majority of participants from all continents thought that clinical immunology includes routine laboratory procedures and research (**Table 2**).

**Table 2:** Geographical distribution of opinions regarding the WHO definition of Clinical Immunology.

	Africa	America	Asia	Europe	Oceania	Total	p
Too broad (%)	54.1	58.95	30.68	31.62	29.17	59.3%	<0.0001 ^a^
Incomplete (%)	22.95	33.68	19.32	19.85	20.83	23.0%	0.08 ^a^
Exclusive to patient care (%)	24.59	24.21	24.32	20	56.52	37.6%	<0.0001 ^a^
Includes routine lab (%)	90.16	84.21	88.64	83.82	70.83	84.7%	0.002 ^a^
Includes research (%)	96.72	97.89	98.86	89.71	87.5	94.6%	0.0007 ^a^
A CI is a physician (%)	80.33	88.42	73.86	69.85	100	77.2%	<0.0001 ^a^

^a^ Chi square. CI, Clinical Immunologist.

## Discussion

This study presents for the first time the results of a world-wide questionnaire that gathered the opinion of more than five hundred clinical immunologists about their specialty. Overall, our results suggest that clinical immunologists are mostly physicians that care for children and/or adults and are mostly involved in the treatment of inborn errors of immunity and allergic diseases. As expected, their clinical practice closely mirrors their clinical training and other conditions where immunology plays a central role (e.g., cancer immunotherapy, etc.) are not consistently considered by the specialty, neither during training nor professional practice.

The survey was applied to clinical immunologists belonging to a wide range of age groups and, consequently, with a wide range of experience. In this sense, we believe that our results reflect the overall understanding about our specialty around the world. Because the questionnaire was distributed through professional FOCIS and IUIS member associations, it was anticipated to mostly have reached professionals working mainly in clinical practice and other related settings, such as clinical or research laboratories. In fact, a fraction of respondents declared that their main responsibility was related to research. A comparison between respondents who are directly involved in patient care and those who do not see patients on a regular basis, evidenced that these two groups of professionals differ in their understanding of Clinical Immunology. Clinicians thought more often that Clinical Immunology is a medical specialty in which the clinical immunologist is a physician and consequently, Clinical Immunology is limited to clinical practice and the WHO definition is too broad. We believe that this interpretation results from the somewhat subtle difference between a *clinical immunologist*, a professional that practices Clinical Immunology, and *Clinical Immunology* as a medical field. In most countries, a clinical immunologist is indeed a physician specialized in caring for patients afflicted by immune mediated disorders, mainly all types of inborn errors of immunity or allergic diseases. In contrast, Clinical Immunology is a broad field in which not only clinical immunologists are involved, but also basic and clinical researchers, as well as laboratory personnel.

Along the same lines, we believe that the expansion of immunology into a broad variety of medical fields has undoubtedly increased the breadth of Clinical Immunology. However, we did not observe an analogous expansion in the practice or teaching of clinical immunologists. In other words, clinical immunologists are still mostly trained to care for patients with allergic diseases and inborn errors of immunity. The growing number and variety of these genetic diseases has increased the complexity of the clinical practice and research in Clinical Immunology in such an extent that the field must acknowledge the need of assembling multidisciplinary teams of clinicians and researchers with complementary expertise (13–16). Clinical immunologists must work side by side with other specialists during the care of their patients and during the care of patients that traditionally belong to other specialties (17). For example, the use of immune checkpoint blockade induces a large variety of autoimmune and inflammatory syndromes, and the use of immunosuppressive drugs imposes acquired immunodeficiencies where the expertise of clinical immunologists is most likely useful (10, 18).

In this sense, though we believe that the 1993 WHO definition of Clinical Immunology is still valid, the “diseases or disease processes resulting from disordered immunological mechanisms” have greatly increased and thus, while the definition is still accurate, the breadth of the field has considerably grown. Therefore, we suggest adding the following sentence to the definition of Clinical Immunology: “The complexity of diagnosis and treatment of some patients implies a close interaction with basic immunology, genetics, gene and cell therapy.”

The Clinical Immunology Committee of the IUIS is preparing a new survey that will explore in detail how Clinical Immunology is taught around the world, because the results of this questionnaire suggest that the field has grown in a disproportionate manner in comparison with the breadth of training of clinical immunologists.

In conclusion, our results suggest that our specialty has grown, but our understanding of our role as clinical immunologists remains mostly defined, notwithstanding the ever-expanding role of immunology in fields such as transplantation and oncology. Because novel disease paradigms place immunological processes in a central position and a wealth of new therapies are based on the modification of immune function, we believe that clinical immunologists are in a key position to embrace the opportunity these changes represent and to participate in the study, diagnosis, and management of patients with diseases different from the ones traditionally treated by them, as key immune components are now recognized in diseases formerly not associated with monogenic defects in genes that control immune function.

## Data Availability

The raw data supporting the conclusions of this article will be made available by the authors, without undue reservation.
