# Supplementary material for: Definition of clinical immunology around the globe
Source: Front Immunol. 2025 Jan 28;16:1483391. doi: 10.3389/fimmu.2025.1483391 (PMC11810920; doi:10.3389/fimmu.2025.1483391)
Supplement: Supplementary file 1 [file DataSheet1.pdf]

Definition of *Clinical Immunology* around the globe

This questionnaire represents an effort of the Clinical Immunology Committee of the International Union of Immunological Societies (IUIS) to understand how *Clinical Immunology* is perceived around the world.

Our questions cover two aspects: (1) you, as a clinical immunologist; (2) in your country, how are clinical immunology training and activities organized.

The results of this questionnaire will be presented during a round-table at the IUIS Congress in Cape Town, in December 2023.

Thank you for your time!

Tineke Cantaert, Alberto Pinzón, José C. Crispín, Pierre Miossec.

If you have any questions or comments, please email José C. Crispín (jccrispin@tec.mx) or Pierre Miossec (pierre.miossec@univ-lyon1.fr).

* Required

1. Name

2. Email address

3. Which age group do you belong in? *

Mark only one oval.

- <30
- 30-40
- 40-50
- 50-60
- 60-70
- >70
- I'd rather not answer

4. What is your gender? *

Mark only one oval.

- Female
- Male
- Non-binary
- I'd rather not answer

5. In which country were you born? *

6. In which country did you train? *

7. Do you hold an M.D. degree or equivalent (e.g. M.B.B.S.)?

Mark only one oval.

- Yes
- No

8. Did your training include residency? *

Mark only one oval.

- Yes
- No

9. Which residency?

____

10. Did your training include fellowship? *

Mark only one oval.

- Yes
- No

11. Which fellowship?

____

12. Do you hold a Ph.D. degree? *

Mark only one oval.

- Yes
- No

13. In which area is your Ph.D.?

____

14. Have you completed your clinical training? *

Mark only one oval.

- Yes
- No

15. In which year did you complete your training?

____

16. Are you directly involved in patient care? *

Mark only one oval.

- Yes
- No

17. Does your work include clinical/routine laboratory? *

Mark only one oval.

- Yes
- No

18. Which type of patients do you treat?

Mark only one oval.

- ( ) Children
- ( ) Adults
- ( ) Both

19. In which country do you practice?

____

20. How is your time distributed? *

Check all that apply.

	0	1-24%	25-49%	50-74%	75-99%	100%
**Clinical care**	☐	☐	☐	☐	☐	☐
**Clinical/routine laboratory**	☐	☐	☐	☐	☐	☐
**Basic research**	☐	☐	☐	☐	☐	☐
**Clinical research**	☐	☐	☐	☐	☐	☐

21. Clinical immunology is defined by the WHO as *a clinical and laboratory discipline dealing with the study, diagnosis, and management of patients with diseases or disease processes resulting from disordered immunological mechanisms, and conditions in which immunological manipulation form an important part of therapy and/or prevention.* *

Mark only one oval.

- ( ) Completely agree
- ( ) Agree
- ( ) Neutral
- ( ) Disagree
- ( ) Completely disagree

22. Do you believe the WHO definition is too broad? *

Mark only one oval.

- ( ) Yes
- ( ) No

23. Do you believe it is incomplete? *

Mark only one oval.

- ( ) Yes
- ( ) No

24. Do you believe *Clinical Immunology* refers exclusively to the practice of <u>clinical care</u>? *

Mark only one oval.

- ( ) Yes
- ( ) No

25. Do you believe that *Clinical Immunology* includes the practice of clinical/routine laboratory? *

Mark only one oval.

- Yes
- No

26. Do you believe that *Clinical Immunology* includes research oriented towards immunological processes and diseases? *

Mark only one oval.

- Yes
- No

27. Do you assume that a *Clinical Immunologist* is a physician that cares for patients? *

Mark only one oval.

- Yes
- No

28. In the country where you practice, in which conditions is a *Clinical Immunologist* involved in patient care and in which role? *

Check all that apply.

	Main physician	Consultant	Not involved
**Allergic diseases, anaphylaxia, hereditary angioedema, urticaria**	☐	☐	☐
**Asthma**	☐	☐	☐
**Autoimmune rheumatologic diseases**	☐	☐	☐
**Autoimmune diseases**	☐	☐	☐
**Infectious diseases**	☐	☐	☐
**Inborn errors of immunity**	☐	☐	☐
**Secondary immunodeficiencies**	☐	☐	☐
**Solid organ transplants**	☐	☐	☐
**Bone marrow transplants**	☐	☐	☐
**Cancer**	☐	☐	☐
**Immunotherapy**	☐	☐	☐

29. In the country where you practice, is there a dedicated residency or fellowship program to train as a Clinical Immunologist? *

Mark only one oval.

- Yes
- No

30. If so, is it shared with other specialty, for example Allergic Diseases?

Mark only one oval.

- Yes
- No

31. If so, which conditions are considered in the curriculum of the *Clinical Immunology fellowship*? *

Check all that apply.

	Yes	No
**Allergic diseases, anaphylaxia, hereditary angioedema, urticaria**	☐	☐
**Asthma**	☐	☐
**Autoimmune rheumatologic diseases**	☐	☐
**Autoimmune diseases**	☐	☐
**Infectious diseases**	☐	☐
**Inborn errors of immunity**	☐	☐
**Secondary immunodeficiencies**	☐	☐
**Solid organ transplants**	☐	☐
**Bone marrow transplants**	☐	☐
**Cancer**	☐	☐
**Immunotherapy**	☐	☐

32. In the country where you practice, is there an official examination (e.g. board exam) that regulates *Clinical Immunologists* (physicians)? *

Mark only one oval.

- Yes
- No

33. In the country where you practice, is there an official examination (e.g. board exam) that regulates *Clinical Immunologists* (laboratory personnel)? *

Mark only one oval.

- Yes
- No

34. In the country where you practice, are there *Professors of Clinical Immunology*? *

Mark only one oval.

- Yes
- No

35. Thank you very much for answering this questionnaire!
Please leave us any additional comments or suggestions.

This content is neither created nor endorsed by Google.

Google Forms
